# Multi-Modality Fusion and Tumor Sub-Component Relationship Ensemble Network for Brain Tumor Segmentation

**DOI:** 10.3390/bioengineering12020159

**Published:** 2025-02-06

**Authors:** Jinyan Zhou, Shuwen Wang, Hao Wang, Yaxue Li, Xiang Li

**Affiliations:** 1 Basic Medical College, Heilongjiang University of Chinese Medicine, Harbin 150040, China; zhoujinyan1797@163.com (J.Z.); swwang_3236@163.com (S.W.); 2 Department of Control Science and Engineering, Harbin Institute of Technology, Harbin 150001, China; haowangzzuer@163.com; 3 College of Information Science and Engineering, Northeastern University, Shenyang 110819, China; 4 Hebei Key Laboratory of Micro-Nano Precision Optical Sensing and Measurement Technology, Qinhuangdao 066004, China

**Keywords:** gliomas, brain tumor segmentation, feature recalibration network, multi-modality

## Abstract

Deep learning technology has been widely used in brain tumor segmentation with multi-modality magnetic resonance imaging, helping doctors achieve faster and more accurate diagnoses. Previous studies have demonstrated that the weighted fusion segmentation method effectively extracts modality importance, laying a solid foundation for multi-modality magnetic resonance imaging segmentation. However, the challenge of fusing multi-modality features with single-modality features remains unresolved, which motivated us to explore an effective fusion solution. We propose a multi-modality and single-modality feature recalibration network for magnetic resonance imaging brain tumor segmentation. Specifically, we designed a dual recalibration module that achieves accurate feature calibration by integrating the complementary features of multi-modality with the specific features of a single modality. Experimental results on the BraTS 2018 dataset showed that the proposed method outperformed existing multi-modal network methods across multiple evaluation metrics, with spatial recalibration significantly improving the results, including Dice score increases of 1.7%, 0.5%, and 1.6% for the enhanced tumor core, whole tumor, and tumor core regions, respectively.

## 1. Introduction

Glioma is the most common primary malignant tumor of the central nervous system, accounting for more than 50% of all malignant tumors [1]. The tumors have a high recurrence rate after surgery, poor prognosis, and low survival rates [2]. Gliomas are usually divided into grades I and II, and III and IV, where grades I and II are classified as low-grade gliomas (LGGs) and the last two grades are classified as high-grade gliomas (HGGs) [3]. The internal structure of gliomas is notably complex, encompassing regions such as edema (ED), necrosis (NCR), enhancing tumor (ET), and non-enhancing tumor core (NET) [4,5,6]. Once gliomas are diagnosed, surgical removal can effectively reduce tumor size [7,8]. This is typically followed by comprehensive treatments, including radiotherapy, chemotherapy, and targeted drug therapies. Given the high recurrence rate and variable prognosis of gliomas, patients require personalized, multidisciplinary treatment plans [9,10]. With the increasing pace of modern life and rising levels of stress, the incidence of gliomas has been steadily growing, posing a significant threat to public health.

Diagnosing gliomas involves multiple examinations, including magnetic resonance imaging (MRI), computed tomography, biochemical tests, neurological assessments, and biopsies [11]. Among these, MRI is the gold standard for glioma evaluation [12]. The diverse imaging characteristics across different body regions make it difficult to fully understand complete anatomical features. To address these issues, this study introduces a multi-modality and single-modality feature recalibration network (MSFR-Net), a model designed for precise brain tumor segmentation. Multi-modality (MM) MRI, commonly used in clinical practice for brain tumor analysis, typically includes four imaging modalities: T1/T2-weighted imaging (T1WI/T2WI), contrast-enhanced T1-contrast imaging (T1c), and fluid-attenuated inversion recovery (Flair) [13,14]. The different modalities provide unique biological insights into various physiological conditions, enabling a comprehensive understanding of tumor characteristics. For instance, T1WI effectively highlights fat-rich tissues, such as subcutaneous fat and bone marrow, as high-signal regions, aiding in the clear visualization of these structures [15]. Conversely, tumor regions appear as low-signal areas, facilitating differentiation between healthy brain tissue and the tumor region as a whole, though the internal tumor substructures are less distinct. T2WI and Flair imaging are particularly suited for visualizing ED, which manifests as high-signal regions [16]. T1c enhances the contrast between the tumor core and necrotic regions, providing clearer visualization of tumor boundaries. The combination of MM MRI modalities offers radiologists a wealth of information on tumor morphology and volume, playing a critical role in tumor grading, treatment planning, and surgical preparation.

## 2. Related Work

The high dimensionality, heterogeneity, and unstructured nature of biomedical data, coupled with insufficient data annotation, limit the effective training and generalization capabilities of deep learning models, especially in medical image analysis. The limitations of traditional methods and deep learning in processing complex data are as follows: Traditional methods have difficulty in efficiently extracting the features of complex data, and although deep learning offers the potential for end-to-end learning, it still faces technical and application challenges in dealing with data imbalances, enhancing model robustness, and improving accuracy. In response to these challenges, some recent research results and technical contributions are presented. Early approaches to BT segmentation predominantly focused on feature extraction and classifier design. Feature extraction typically involves morphological, texture, and intensity-based features. Researchers often employed morphological and computer graphics techniques to manually describe tumor characteristics [17,18]. With the development of artificial intelligence technology, the application of deep learning technology in the field of medical image segmentation has attracted widespread attention from scholars [19]. Within this trend, image classification and segmentation methods based on convolutional neural networks (CNN) have become increasingly popular [20,21,22,23]. A brain tumor classification method based on a generative adversarial network was proposed, and the classification performance was significantly improved through data augmentation and model optimization [24]. Reference [25] proposed a deep-learning-based auxiliary diagnosis system, which uses data enhancement, denoising technology, and a CNN to provide a new solution for the early diagnosis of brain glioma. A brain tumor segmentation network based on an encoder–decoder architecture was proposed, which innovatively combines a sparse dynamic module and a multi-layer edge feature fusion module to effectively improve segmentation accuracy [26]. CNNs have become leading tools for BT segmentation, outperforming traditional methods in both accuracy and speed [27,28]. CNNs are highly effective at extracting deep, hierarchical features from images and have demonstrated outstanding performance in MM brain tumor segmentation. Reference [29] introduced a multi-category BT segmentation algorithm based on 3D brain imaging, which effectively solved the problems caused by the complexity of MM MRI data and the variability in BT regions. Reference [30] proposed a Transformer-based cross-modal shared learning network that effectively extracted MM CF for BT segmentation. Reference [31] presented an innovative modality pairing learning method for BT segmentation, employing parallel branches and a 3D U-Net backbone to efficiently fuse multimodal MRI features and capture complex inter-modal relationships.

In [32], average Dice scores (DC) of 91.2%, 81.8%, and 88.3% for whole tumor (WT), enhancing tumor (ET), and tumor core (TC) segmentation, respectively, were achieved using a simple CNN on the BraTS 2018 dataset with 200 randomly selected samples. While this work demonstrated promising results, it relied on an incomplete dataset. By contrast, other studies have utilized the complete dataset for validation. Ranjbarzadeh et al. [33] proposed a cascaded convolutional neural network (C-CNN) that extracts features from both local and global perspectives, achieving DC scores of 80.1%, 90.6%, and 84.5% for ET, WT, and TC segmentation, respectively. Chen et al. [34] employed a dilated multi-fiber network (DMFNet) to construct multi-scale feature representations, also achieving DC scores of 80.1%, 90.6%, and 84.5% for ET, WT, and TC regions. Sun et al. [35] used a set of three distinct 3D CNN models fused for tumor segmentation, improving the performance and minimizing bias through majority voting, resulting in DC scores of 80.5%, 90.4%, and 84.9%, respectively. An enhanced U-Net model was proposed for multimodal brain tumor MRI segmentation, achieving DC scores of 89.0%, 82.0%, and 79.0% for WT, TC, and ET on the training set [36]. Zhang [37] utilized deep residual learning combined with multimodal image feature fusion to improve BT image segmentation accuracy, achieving DC scores of 83.3%, 89.1%, and 91.4% for the ET, TC, and WT regions, respectively. Additionally, an interactive modality deep learning method was developed to extract discriminative BT information from MRI data, achieving DC scores of 90.9%, 85.4%, and 80.1% for the ET, TC, and WT regions [38]. A literature survey of related works is shown in Table 1.

**Figure 1:**
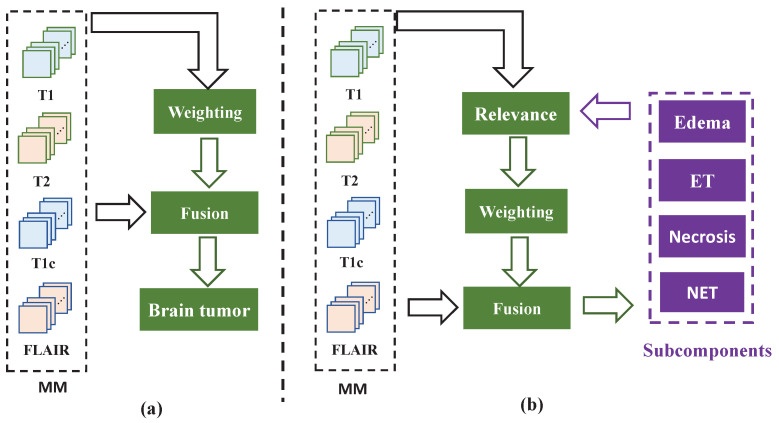
(**a**) Structure diagram of the existing methods. These methods generally involve either direct fusion of MM MRI data or weighted fusion of MM features. (**b**) Structure diagram of the proposed method. This method fuses the special features extracted by the SMN with the features extracted by the MMN.

**Figure 2:**
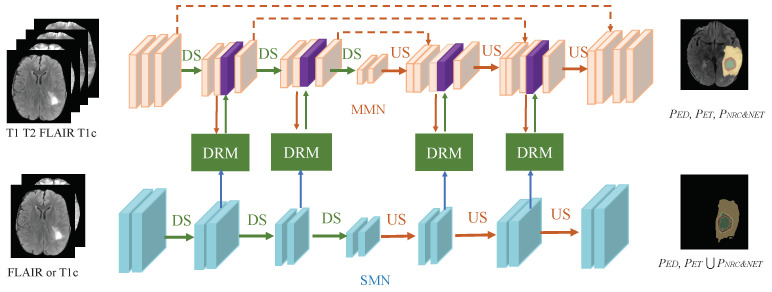
The proposed MMN and SMN aggregation structure. The MMN and SMN extract dedicated features and complementary features, respectively. The dual recalibration module (DRM) strengthens these two features and then provides the result to the MMN.

Previous studies have successfully demonstrated that weighted fusion segmentation methods can obtain modality importance information, laying a solid foundation for MM medical image segmentation. However, to the best of our knowledge, challenges such as multi-modality features and single-modality features remain unsolved, which motivated us to explore effective solutions for the fusion of multi-modality features and single-modality features. Figure 1a illustrates the structure of existing methods, which primarily focus on leveraging MM information fusion to extract meaningful features. These methods generally involve either direct fusion of MM MRI data or weighted fusion of MM features. These methods mainly focus on the integration of MM information, without fully integrating the relationship between single-modality and tumor subcomponents. This special relationship is specifically manifested in that different modality images have different sensitivities to different components inside brain tumors, and there is a specific correspondence between the imaging effect and different components. From the imaging characteristics, the tumor area in the T1WI shows a low pixel intensity, and the distinction between healthy tissue and the overall tumor area is relatively clear, but the distinction between subcomponents inside the tumor area is not obvious. T2WI and Flair images show good edema areas, and the boundary between the overall edema area and the TC is clear. The boundary between the peripheral edema area and the entire tumor in the T1c image is not clear, but there is good contrast between the ETC and NCR. A multi-modality network (MMN) learns to fuse features from MM images under the supervision of all segmented subcomponents, while a single-modality network (SMN) can capture the relationship between modality features and SM and extract specific features of SM images. The existing literature has not fully explored whether the dedicated features captured by the SMN can be used to fuse the fusion features extracted by the MMN to improve the performance of the MMN. As shown in Figure 1b, the unique contribution of our approach is the fusion of the specialized features extracted by SMN and features extracted by the MMN to improve the performance of the MMN. The framework is shown in Figure 2, and we propose incorporating the special relationship between SM features and tumor subcomponents into the MM fusion process to improve the performance of the image segmentation algorithms of the MMN. Specifically, we adopt an MM and SM feature recalibration network (MSFR-Net) to preserve the MMN information, while integrating the SMN features for tumor segmentation. This fusion of multimodal and SM information improves the diagnostic accuracy and BT segmentation performance of the MMN. The MMN and SMN run in parallel from input to output and are connected by a DRM, ensuring that multimodal information is preserved throughout the segmentation process, while not ignoring SM information. The contributions of this work are as follows:

1. This paper integrates MM and SM features to achieve effective brain tumor segmentation.
2. A DRM is introduced into the network to fuse the information of the MMN and SMN, enabling comprehensive feature calibration.
3. The training process of the MMN incorporates tumor subcomponent information emphasized by the SMN, significantly enhancing the segmentation accuracy of the MMN.

## 3. Method

### 3.1. Overview of the Proposed Method

In the task of BT segmentation, MM MRI includes four imaging modalities: T1WI, T2WI, T1c, and Flair, represented as $\left\{D_{T1},D_{T2},D_{T1c},D_{Flair}\right\}$. The internal structure of brain tumors is highly complex, with subcomponents primarily consisting of ED, ET, NCR, and NET, denoted as $\left\{P_{ED},P_{ET},P_{NRC\&NET}\right\}$, respectively. Figure 2 illustrates the network architecture, where the network is designed to extract CF and SF, respectively. The DRM serves to fuse these features and further integrate them into the MMN for enhanced performance. Both MMN and SMN include two processes: upsampling (US) and downsampling (DS). This paper introduces the MMFR-Net, which extracts CF across all modalities via the MMN and SF via the SMN. To achieve this, parallel architectures for the MMN and SMN are constructed. The MMN takes the CF of T1WI, T2WI, T1c, and Flair as input. The training process is supervised using all sub-goals of BT segmentation, and the prediction is formulated as follows:

(1) $$\begin{array}{c} \left\{{\hat{P}}_{ED},{\hat{P}}_{ET},{\hat{P}}_{NRC\&NET}\right\}=f_{MMN}\left(D_{T1},D_{T2},D_{T1c},D_{F}\right) \end{array}$$

where $\left\{{\hat{P}}_{ED},{\hat{P}}_{ET},{\hat{P}}_{NRC\&NET}\right\}$ are the prediction results of the MMN.

With its emphasis on the dedicated features representing the relationship between the SMN and its most relevant tumor sub-target, SM feature extraction offers valuable insights. Leveraging the characteristics of MRI and the radiologists’ annotation process, two specific networks, T1c and Flair, are employed. Flair images provide excellent visualization of edema, making them suitable for training with labels $\left\{P_{ED},P_{ET},P_{NRC\&NET}\right\}$. In this configuration, $P_{ET}$ and $P_{NRC\&NET}$ are combined for Flair network training. On the other hand, T1c enhances the contrast between the tumor core and necrotic tissue by improving the image contrast, making it suitable for training with labels $\left\{P_{ET},P_{NRC\&NET}\right\}$. The learning process of the SMN can be formulated as follows:

(2) $$\left\{{\hat{P}}_{ED},{\hat{P}}_{ET}\cup {\hat{P}}_{NRC\&NET^{\prime }}\right\}=f_{SMN}\left(D_{T1c}\right)$$

(3) $$\left\{{\hat{P}}_{ET},{\hat{P}}_{NRC\&NET}\right\}=f_{SMN}\left(D_{Flair}\right)$$

where $\left\{{\hat{P}}_{ED},{\hat{P}}_{ET},{\hat{P}}_{NRC\&NET}\right\}$ are the prediction results of SMN.

Both the MM and SM networks adopt a 3D U-Net architecture, which consists of encoding and decoding processes. The input to the network comprises four modalities superimposed in the channel dimension. The number of feature map channels (FMC) in the network is set to 32, 64, 128, and 256, respectively. Since 3D convolutional neural networks consume substantial GPU memory, the SM network is designed to focus on specific features and reduce the number of FMCs, thereby minimizing the memory consumption. In the MM network, convolution operations are applied at each basic network layer, complemented by the ReLU activation function and normalization layers to enhance the network sparsity and mitigate overfitting. Four symmetrical DRMs are introduced to process the encoding and decoding stages. SM features, after processing by the DRMs, are fused into the MM network. The use of DRM modules ensures accurate channel dimensions when handling SM and MM features. A lightweight design approach is adopted to reduce the model’s parameter count and overall complexity. By structuring convolutional layers to progressively increase the number of channels, the MM network dynamically adjusts its feature channel capacity to accommodate complex representations.

### 3.2. Dual DRM Module and Loss Function

The dual DRM module is designed to adjust and optimize the processing mechanism within the network. It recalibrates features through two distinct processes, spatial recalibration (SR) and channel recalibration (CR), to achieve more accurate and optimized results. Directly connecting MM and SM networks in series may compromise the network’s effectiveness in extracting tumor-related features. To address this issue, the dual DRM module integrates SR and CR to enhance the feature extraction and fusion.

Specifically, SR refers to the adjustment or transformation of spatial features in input data, aiming to extract and utilize feature information more effectively within deep learning frameworks. Let $F_{MMN}$, $F_{T1c}$, and $F_{Flair}$ denote the features extracted by the MM, T1c, and Flair networks, respectively, and $\left\{F_{MMN},F_{T1c},F_{Flair}\right\}\in R^{O\times P\times Q\times R}$. Where *O*, *P*, *Q*, and *R* indicate the length, width, height, and number of channels of the characteristic tensor, respectively. Since this step only considers the spatial relationship of the three features, the number of channels is compressed through a convolution layer with a kernel size of 1×1×1 to obtain the spatial weight $W_{MMN}^{SR}$, $W_{T1c}^{SR}$, and $W_{Flair}^{SR}$, with the O×P×Q dimension. The next three are stacked in the channel dimension and the weights of the space are calculated using the Softmax function. This process can be expressed as follows:

(4a) $$W_{MMN}^{SR}=f_{\mathrm{Conv}\;}\left(F_{MMN}\right)$$

(4b) $$W_{T1c}^{SR}=f_{\mathrm{Conv}\;}\left(F_{T1c}\right)$$

(4c) $$W_{Flair}^{SR}=f_{\mathrm{Conv}\;}\left(F_{Flair}\right)$$

where $\begin{array}{c} W_{MMN}^{SR},W_{T1c}^{SR},W_{\mathrm{Flair}\;}^{SR} \end{array}$ are the spatial feature. The weight is calculated using the Softmax function.

(5) $$\left\{W_{MMN}^{SR^{\prime }},W_{T1c}^{SR^{\prime }},W_{Flair}^{SR^{\prime }}\right\}=f_{\mathrm{Softmax}\;}\left(W_{MMN}^{SR}⊙W_{T1c}^{SR}⊙W_{Flair}^{SR}\right)$$

where ⊙ represents a connection between two channels. $W_{MMN}^{SR^{\prime }},W_{T1c}^{SR^{\prime }},W_{Flair}^{SR^{\prime }}$ are tensors of dimensions P×Q×R. $f_{\mathrm{Softmax}\;}\left(*\right)$ represents the Softmax kernel function. Then, the weighted sum of these features is used to further obtain the SR features.

(6a) $$F_{MMN}^{SR}=W_{MMN}^{SR^{\prime }}\times F_{MMN}$$

(6b) $$F_{T1c}^{SR}=W_{T1c}^{SR^{\prime }}\times F_{T1c}$$

(6c) $$F_{Flair}^{SR}=W_{Flair}^{SR^{\prime }}\times F_{Flair}$$

where $F_{MMN}^{SR}$, $F_{T1c}^{SR}$, $F_{Flair}^{SR}$ are spatial recalibration features.

CR is a common strategy in deep learning, designed to adjust the relationships among FMC and enhance a network’s feature learning capabilities. The primary objective of CR is to dynamically modify the feature channels within the network, assigning different weights to variations in the input data’s features. This mechanism improves the network’s ability to recognize and process diverse features, thereby enhancing the representation of tumor-related characteristics. Each channel in the network’s feature map can be interpreted as a response to a specific type of feature. To achieve effective recalibration, the global average pooling (GAP) method is employed to extract and retain only channel-specific information, enabling more focused and efficient feature processing.

(7a) $$W_{MMN}^{CR}=f_{GAP}\left(F_{MMN}^{CR}\right)$$

(7b) $$W_{T1c}^{CR}=f_{GAP}\left(F_{T1c}^{CR}\right)$$

(7c) $$W_{Flair}^{CR}=f_{GAP}\left(F_{Flair}^{CR}\right)$$

where $\begin{array}{c} W_{MMN}^{CR},W_{T1c}^{CR},W_{\mathrm{Flair}\;}^{CR} \end{array}$ are the channel information. Then, the sigmoid function is used to obtain the weight.

(8a) $$W^{CR}=W_{MMN}^{CR}⊙W_{T1c}^{CR}⊙W_{Flair}^{CR}$$

(8b) $$W_{MMN}^{CR^{\prime }}=f_{\mathrm{Sigmoid}\;}\left(f_{FC}\left(W^{CR}\right)\right)$$

(8c) $$W_{T1c}^{CR^{\prime }}=f_{\mathrm{Sigmoid}\;}\left(f_{FC}\left(W^{CR}\right)\right)$$

(8d) $$W_{Flair}^{CR^{\prime }}=f_{\mathrm{Sigmoid}\;}\left(f_{FC}\left(W^{CR}\right)\right)$$

where $W_{MMN}^{CR^{\prime }}$$W_{T1c}^{CR^{\prime }}$ and $W_{Flair}^{CR^{\prime }}$ are channel weights.

Finally, multiply the channel weights with the eigenvalues to complete the channel reset calibration:

(9a) $$F_{MMN}^{CR}=W_{MMN}^{CR^{\prime }}\times F_{MMN}^{CR}$$

(9b) $$F_{T1c}^{RC}=W_{T1c}^{CR^{\prime }}\times F_{T1c}^{CR}$$

(9c) $$F_{Flair}^{CR}=W_{Flair}^{CR^{\prime }}\times F_{Flair}^{RC}$$

where $F_{MMN}^{CR}$, $F_{T1c}^{RC}$, $F_{Flair}^{CR}$ are features after channel calibration.

Finally, the output of the entire DRM can be expressed as

(10) $$F_{out}=F_{MMN}^{CR}+F_{T1c}^{CR}+F_{Flair}^{CR}$$

To mitigate the issue of class imbalance, the proposed network is optimized using a combination of Dice loss and cross-entropy (CE) loss. The formulation of the Dice loss is as follows:

(11) $$L_{\mathrm{Dice}\;}=1-\frac{2}{N_{p}}\sum_{j=1}^{N_{p}}\frac{\sum_{i=1}^{M}\delta \left({\hat{P}}_{j}^{j}\right)P_{j}^{i}}{\sum_{i=1}^{M}\delta \left(P_{j}^{j}\right)+\sum_{i=1}^{M}P_{j}^{i}}$$

where $N_{p}$ denotes the number of tumor subcomponent categories; and *M*, δ, and ${\hat{P}}_{j}^{i}$ represent the number of voxels in the input image, the softmax activation function, and the predicted output, respectively. $P_{j}^{i}$ is the ground truth segmentation value. To ensure a stable training process and reduce the instability associated with using Dice loss alone, cross-entropy (CE) loss is integrated into the overall loss function. The CE loss is expressed as

(12) $$L_{CE}=-\frac{1}{N_{p}M}\sum_{j=1}^{N_{p}}\sum_{i=1}^{M}P_{j}^{i}\log\delta \left(P_{j}^{j}\right)$$

The total loss $L_{T}$ is constructed by combining the MMN loss ($L_{MMN}$) and the SMN loss ($L_{SMN}$), which are weighted and summed. The overall network loss function is defined as

(13) $$L_{T}=\lambda L_{MMN}+\frac{1-\lambda }{2}\left(L_{SMN-T1C}+L_{SMN-Flair}\right)$$

where λ is a weighting factor that balances the contributions of the MMN loss and the SMN loss.

## 4. Experimental Setup

### 4.1. Experimental Datasets

We evaluated the proposed method on the BraTS 2018 [39] and BraTS 2015 [16] datasets. The BraTS 2018 dataset includes MRI images from 285 glioma patients, comprising 75 LGGs and 210 HGGs in the training set, 191 cases in the test set, and 66 cases of gliomas with unknown grades in the validation set. The BraTS 2015 dataset contains 54 LGGs and 220 HGGs in the training set, and 110 gliomas of unknown grade in the test set. These datasets have been widely used to validate advanced algorithms, providing a standardized benchmark to test and compare the proposed method against existing approaches.

### 4.2. Training Data

The data in both datasets are 3D MRI volume data with a size of 155 × 240 × 240. Prior to training, necessary preparations were carried out, including preprocessing the images. Voxel intensities were normalized to follow a standard normal distribution. Voxel blocks of size 96 × 128 × 128 were randomly cropped from the entire image as training samples. To enhance the model robustness, the samples underwent random rotations, scaling, elastic deformations, and gamma corrections. In each iteration, 300 voxel blocks were randomly selected for cropping and batch training. Hyperparameter tuning was performed using a grid search strategy within a predefined parameter space to optimize the model performance. The learning rate was evaluated at three levels: 2 × $10^{-5}$, 1 × $10^{-4}$, and 2 × $10^{-4}$. Weight decay values of 1 × $10^{-5}$, 2 × $10^{-5}$, and 1 × $10^{-4}$ were tested. Batch sizes of 2, 4, and 8 were analyzed for their effect on the model performance. Additionally, three optimizers, SGD, Adam, and AdamW, were compared, with Adam demonstrating the best overall performance. According to the grid search results, the final hyperparameters were set to 100 epochs and the batch size was 4. The initial learning rate was set to 1 × $10^{-4}$, and the weight decay was assigned to 2 × $10^{-5}$. The entire network framework was implemented using the PyTorch framework 1.11.0.

### 4.3. Evaluation Metrics

The test process adhered to the evaluation metrics of the official BraTS competition, namely the DC, Hausdorff distance (${\mathrm{HD}}_{95}$), and positive predictive value (PPV).

(14a) $$\;\mathrm{Dice}\;=\frac{2N_{TP}}{N_{FP}+2N_{TP}+N_{FN}}$$

(14b) $$Sensitivity=\frac{N_{TP}}{N_{TP}+N_{FN}}$$

(14c) $$PPV=\frac{N_{TP}}{N_{TP}+N_{FP}}$$

(14d) $$\mathrm{Haus}\left(P,T\right)=\max\left\{\underset{p\in P}{\sup}\underset{t\in T}{\inf}dis\left(p,t\right),\underset{t\in T}{\sup}\underset{p\in P}{\inf}dis\left(t,p\right)\right\}$$

where $T_{TP}$ and $T_{FP}$ are the number of true and false positives. $T_{FN}$ is false negative voxels. In the Hove distance, *P* and *T* are split surfaces and real value surfaces, and *p* and *t* are points on *P* and *T*. The sup and inf are the upper boundary and the lower boundary, respectively. dis(∗) is the distance function.

The DC is commonly used to measure the similarity between predicted and ground truth segmentations. It is a widely recognized metric for evaluating model performance, primarily assessing the consistency between predicted segmentation results and actual labels. ${\mathrm{HD}}_{95}$ represents the 95th percentile of the HD calculated from the set of point pairs in the segmented pixel or voxel space. After spatial scale transformation, ${\mathrm{HD}}_{95}$ is expressed in physical units as millimeters, providing a clinically meaningful evaluation of segmentation accuracy. PPV, also known as precision, measures the proportion of correctly predicted positive pixels or voxels among all predicted positives. This metric reflects the reliability of the model in identifying tumor regions without overestimating their extent, which is particularly important in medical image analysis. Together, these metrics provide a comprehensive evaluation of segmentation performance.

## 5. Experimental Results

The experimental results on the BraTS 2018 dataset are presented in Table 2. We used the DC and ${\mathrm{HD}}_{95}$ of ET, WT, and TC as evaluation criteria (CE). The methods that performed well in the competition generally fall into two categories: SM methods and integrated modality approaches. SM methods rely on a single model or algorithm to address the problem or perform data analysis. In contrast, integrated modality methods combine MM and SM features to achieve better results. Integrated modality approaches effectively enhance model stability and prediction accuracy, while reducing the risk of overfitting. Since integrated models leverage the complementary strengths of MM and SM features, their performance is typically superior to that of SM methods alone.

**Figure 3:**
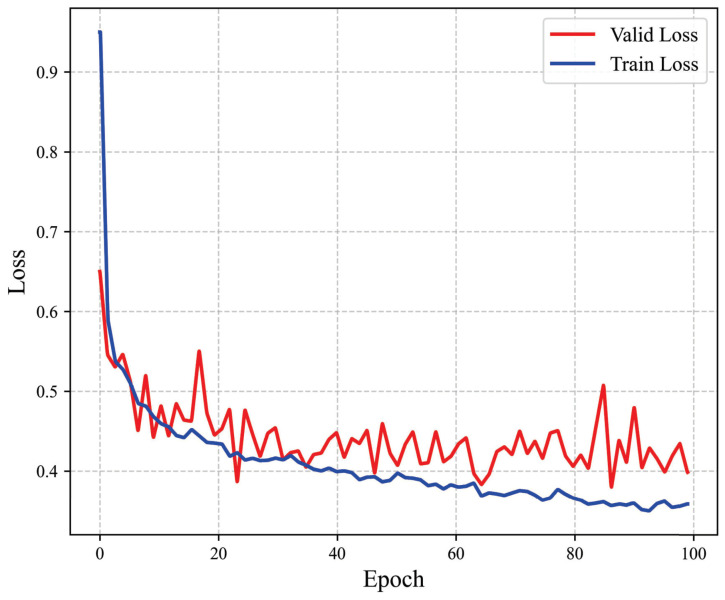
Loss curves of the proposed method on the training and validation sets of the BraTS 2018 dataset. The curves show the loss optimization process within 100 epochs. Both the epoch number and the loss are dimensionless.

Figure 3 shows the loss optimization process within 100 epochs, and we can see that the validation and training loss converged quickly. This shows that the strategy of jointly training and optimizing using multiple loss functions was effective and could achieve good results, without increasing the size of the single-modal network. Table 2 shows the experimental results of the different methods on the BraTS 2018 dataset. According to the competition’s EC, the DC and the 95th percentile of the ${\mathrm{HD}}_{95}$ are reported for the ET and TC. The results indicate that some methods outperformed others in specific performance metrics. For example, Zhou et al. [40] introduced a cross-task guided attention module that excelled in certain aspects. Wang et al. [41] employed a three-level cascade framework for BT segmentation. Isensee et al. [42] proposed a 3D U-Net that achieved the best DC score for the WT. Myronenko et al. [43] utilized an encoder–decoder-based segmentation network and achieved the best ${\mathrm{HD}}_{95}$ score for WT. The proposed method demonstrated notable advantages in the DC and ${\mathrm{HD}}_{95}$ scores for ET and the average metrics, as well as the ${\mathrm{HD}}_{95}$ score for TC. The results highlight that the proposed approach effectively improved the Hausdorff distance, validating the strategy of leveraging the MMN to focus on the entire tumor region and the SMN to target tumor sub-regions.

For BraTS 2015, the DC, PPV, and sensitivity were used as EC, and the results are presented in Table 3. Chen et al. [44] proposed a network to decompose the multi-label brain tumor segmentation problem into multiple binary segmentation tasks. Their method and the methods of the other two papers achieved the highest average PPV score. Kamnitsas et al. [45] adopted a dual-path architecture to process input images at multiple scales, achieving the highest PPV for the TC. Sun et al. [46] proposed a 3D CNN-based automatic BT segmentation method built upon the U-Net architecture, obtaining the best sensitivity for the ET. Isensee et al. [42] developed a robust CNN segmentation algorithm based on U-Net, leading in the ${\mathrm{HD}}_{95}$ score for ET, the PPV score for the WT, and the sensitivity for TC. The proposed method achieved the highest scores in four key metrics: the DC for ET, WT, and TC, as well as the sensitivity for WT, demonstrating superior overall performance. These results indicate that our method provided highly consistent predictions of tumor regions compared to the ground truth.

## 6. Ablation Studies

An ablation study was conducted on the BraTS 2018 dataset during online testing. For the experiment, 10% of the data were randomly selected from the local test set, while the remaining 90% constituted the regional training set. The key aspect of this study lies in integrating CF and dedicated features within the unimodality in MM and recalibrating these features using the DRM module. The ablation study quantitatively evaluated the effects of these two design elements independently. The evaluation metrics were extended to include the DC, PPV, sensitivity, and the ${\mathrm{HD}}_{95}$.

The method constructs SMNs using T1c and Flair, where the labels for the two SMNs correspond to independent tumor subcomponents. This section delves into the effectiveness of integrating MM and SM strategies, with the experimental configurations outlined as follows:

MM only: Retain the MM network, while removing all SM networks.

MM & Flair SM: Incorporate only the Flair network within the SM network.

MM & T1c SM: Incorporate only the T1c network within the SM network.

MM & SM (AL): Set the labels of the SM network to cover all tumor subcomponents, broadening the focus of the SM. In this configuration, the SM network is tasked with representing the ET area, rather than concentrating solely on the most relevant tumor subcomponents (ED, ET, NCR, and NET).

Without dual DRM: Remove the dual DRM module and connect the MM and SM networks directly using convolutional layers.

Only feature space DRM: Retain the feature space DRM, while removing the feature channel DRM.

Only feature channel DRM: Retain the feature channel DRM, while removing the feature space DRM.

### 6.1. The Results of Combining MMN and SMN

Ablation experiments were conducted to evaluate whether integrating the fusion features of the MMN with the dedicated features of a SMN enhanced the MMN performance. The first four rows of Table 4 summarize the experimental results for the MMN and SMN ablation studies. The results demonstrate that combining the SMN with MMN significantly improved the model performance. Among the SMNs, T1c SMN showed the most substantial improvement in MMN performance, with Dice scores increasing by 0.9%, 0.8%, and 3%, and ${\mathrm{HD}}_{95}$ decreasing by 0.564, 0.40, and 0.641 in the ET core, WT, and TC regions, respectively. Notably, the improvements brought by T1c SMN were concentrated in the tumor core region, while SMN-dedicated features enhanced the segmentation of the WT.

### 6.2. Validity of Dual DRM

**Figure 4:**
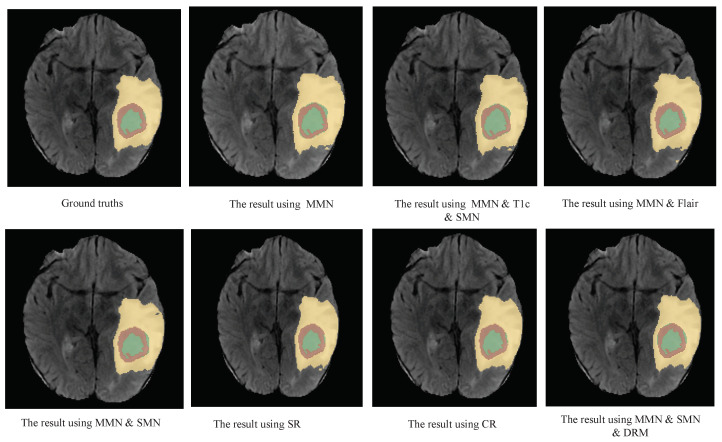
Axial examples of the BT segmentation results on the BraTS 2018 dataset. The results for the first column are ground truth, MMN and SMN (AL). The results for the second column are: MMN only, SR only. The results for the third column are: MMN and T1c and SMN, CR only. The results for the fourth column are: MMN and Flair, MMN and SMN and DRM. Yellow: ED, green: NCR and NET core, brown: ET core. This figure demonstrates the comparative effectiveness of the various configurations in segmenting different tumor subregions in the axial direction.

**Figure 5:**
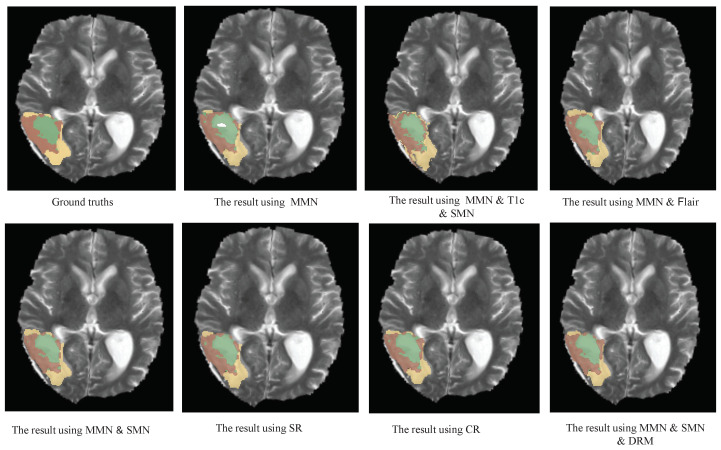
Sagittal examples of the BT segmentation results on the BraTS 2018 dataset. The results for the first column are ground truth, MMN and SMN (AL). The results for the second column are: MMN only, SR only. The results for the third column are: MMN and T1c and SMN, CR only. The results for the fourth column are: MMN and Flair, MMN and SMN and DRM. Yellow: ED, green: NCR and NET core, brown: ET core. This figure demonstrates the comparative effectiveness of the various configurations in segmenting different tumor subregions in the sagittal direction.

**Figure 6:**
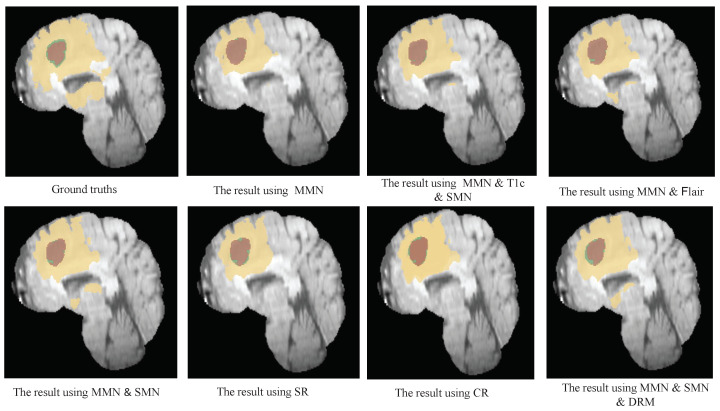
Coronal examples of the BT segmentation results on the BraTS 2018 dataset. The results for the first column are ground truth, MMN and SMN (AL). The results for the second column are MMN only, SR only. The results for the third column are MMN and T1c and SMN, CR only. The results for the fourth column are MMN and Flair, MMN and SMN and DRM. Yellow: ED, green: NCR and NET core, brown: ET core. This figure demonstrates the comparative effectiveness of the various configurations in segmenting the different tumor subregions in the coronal direction.

**Figure 7:**
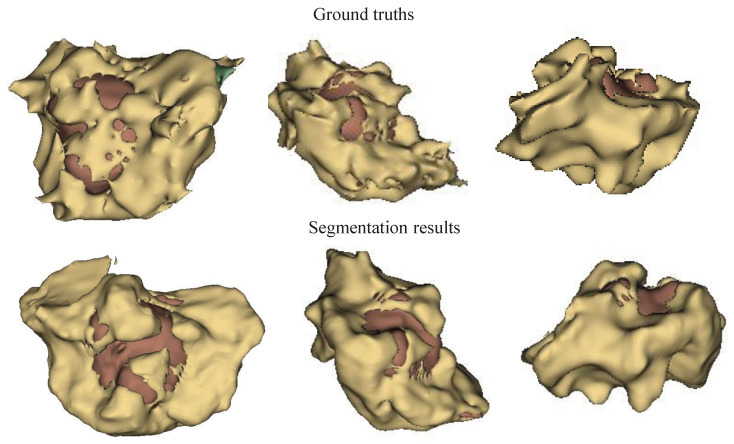
Ground truths and segmentation results in visual perspective 1 using the BraTS 2018 dataset. Yellow: ED, green: NCR and NET core, brown: ET core. This figure provides a clear visual representation of the tumor subregions, aiding in the interpretation and comparison of ground truths and segmentation outcomes.

**Figure 8:**
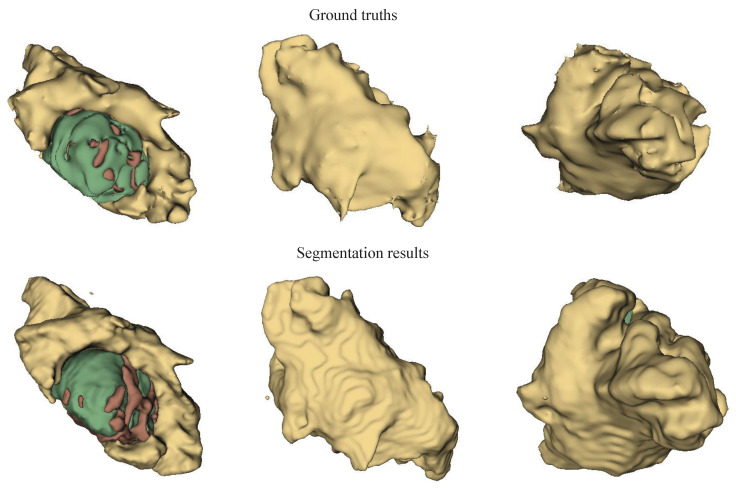
Ground truths and segmentation results in visual perspective 2 using the BraTS 2018 dataset. Yellow: ED, green: NCR and NET core, brown: ET core. This figure highlights the tumor subregions for visual perspective 2, facilitating the evaluation and comparison of segmentation results with ground truths.

**Figure 9:**
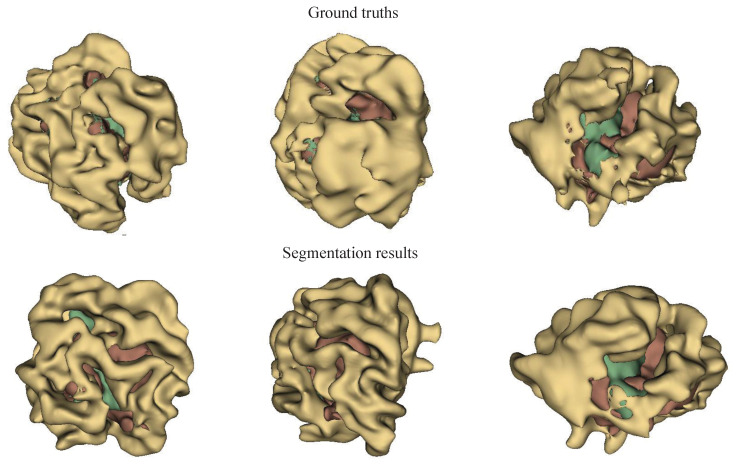
Ground truths and segmentation results in visual perspective 3 using the BraTS 2018 dataset. Yellow: ED, green: NCR and NET core, brown: ET core. This figure provides a clear visual representation of the tumor subregions, aiding in the interpretation and comparison of the ground truths and segmentation outcomes.

Further comparisons between the MMN only and the MMN and SMN with dual recalibration highlighted the positive impact of recalibrated SM networks on segmenting all brain tumor subcomponents. When the MMN and SMN (AL) used labels for all tumor subcomponents, effectively removing the SMN’s relationships with tumor parts, the advantage was primarily reflected in the PPV. This suggests that while the segmentation accuracy improved, the robustness did not increase proportionately. The ablation results for the recalibration module are presented in rows five to seven of Table 4. Comparing the no-DRM and MMN-only setups reveals that directly fusing the MMN and SMN features without recalibration slightly improved the performance, indicating the utility of SMN. However, comparing no DRM and SR only showed that the spatial recalibration significantly contributed to a better performance, with Dice scores improving by 1.7%, 0.5%, and 1.6% for the enhanced tumor core, whole tumor, and tumor core regions, respectively. When comparing SR only and CR only, spatial recalibration demonstrated a more pronounced overall impact. Figure 4, Figure 5 and Figure 6 show the results of the axial, sagittal, and coronal planes, respectively. In order to distinguish the various regions, ED, ET, and NET are marked in green, yellow, and red, respectively. First, the MMN only was particularly prone to under-segmentation or over-segmentation in some error-prone segmentation areas. For instance, the segmentation results of the red area (necrotic and non-enhanced tumor core) in Figure 4 are over-segmented, whereas the red area in Figure 5 is missing. Comparing the results of the MMN only, T1c SMN, and Flair SMN in Figure 6, it can be observed that the T1c SMN and Flair SMN improved the segmentation results, with the T1c SMN showing significant improvement in the red area (necrotic and non-enhanced tumor core). The final network structure combined the advantages of the three features, resulting in a clear segmentation of the boundaries and positions of the sub-components within the brain tumor, achieving results that were very close to the ground truth. From the no-DRM results, it can be seen that, despite the recalibration module not being used, the segmentation performance remained strong, due to the incorporation of features from a single-modality network. Comparing the SR-only and CR-only results, it is evident that they further improved upon the no-DRM baseline. For example, in the green area (edema) and the red area (necrotic and unenhanced tumor core) in the example, the segmentation effects of SR only and CR only are significantly better than those of no DRM. Figure 7, Figure 8 and Figure 9 illustrate the 3D segmentation results alongside the ground truth, highlighting the accuracy of tumor segmentation for both overall and internal regions.

The above ablation experiment results show that the introduction of the DRM enabled the model to capture the key information in the MM images more accurately. Using the DRM to fuse the dedicated features extracted by the SMN with the MM features extracted by the MMN effectively improved the performance of the MMN.

### 6.3. Discussion

To further analyze and compare the performance of existing methods and the proposed method, we can compare them from two perspectives: the number of parameters, and the computing power. The existing methods referenced in [40,48,49] had parameter counts of 13.8 M, 19.3 M, and 23.6 M, respectively. The proposed method had 16.4 M parameters. From the results, we can see that the number of parameters of the method in [40] was less than that of our method. For the training time, the method in reference [50] took 25 h, while our proposed method took 24 h. In terms of testing time, the methods proposed by Kamnitsas et al. [51] and Ma et al. [52] required 35 s and 5 min, respectively, whereas our method achieved a significantly faster testing time of 14 s. Overall, the computational efficiency of our approach is superior and well-suited for clinical applications.

## 7. Conclusions

In this paper, a brain tumor segmentation method was proposed, which fuses the complementary features CF of an MMN and the specific features of an SMN. A DRM unifies the spatial and channel dimension features and achieves the effective fusion of MM and SM features. Compared with the existing multi-modality feature fusion methods, the experimental results on the BraTS 2018 dataset showed that the proposed method achieved better performance for multiple performance indicators. The Dice scores for the ETC, the WT, and the TC region were improved by 1.7%, 0.5%, and 1.6%, respectively. These quantitative results further demonstrate that the MSFR-Net model has certain advantages in handling multi-modality BT segmentation challenges.

The proposed method still has room for improvement in improving the performance of complex multimodal networks, has high computational resource requirements, and lacks sufficient robustness and adaptability to the common modality problems found in clinical settings. In future work, it would also be worth exploring how to further improve the performance of complex multimodal networks (such as C-CNN and 3D CNN) by fusing specialized features extracted from unimodal networks. In addition, we will optimize the MSFR-Net framework through lightweight techniques (such as model pruning and model distillation) to address the computational challenges brought by large-scale parameters and to improve the efficiency and stability of the model under limited hardware resources. Another important direction is to solve the common modality loss problem in clinical environments, and to restore the missing modality information through missing modality recovery methods (such as reversible neural networks and generative adversarial networks), thereby improving the robustness and applicability of the model in actual medical scenarios.

## Figures and Tables

**Table 1:** Summary of related works.

Methods	Dataset	ET			WT			TC		
		DC	Sen	HD_95_	DC	Sen	HD_95_	DC	Sen	HD_95_
CNN [32]	BraTS 2018	0.818	-	-	0.912	-	-	0.883	-	-
C-CNN [33]	BraTS 2018	0.911	0.921	1.669	0.920	0.938	1.427	0.872	0.971	2.408
DMFN [34]	BraTS 2018	0.801	-	3.060	0.906	-	4.660	0.845	-	6.440
3D CNN [35]	BraTS 2018	0.805	0.830	2.777	0.904	0.906	6.327	0.849	0.831	6.327
Improved U-net [36]	BraTS 2018	0.970	0.862	5.982	0.890	0.937	5.468	0.820	0.910	6.846
Fully CNN [37]	BraTS 2021	0.833	-	2.850	0.914	-	5.770	0.891	-	3.97
MMN [38]	BraTS 2018	0.909	-	4.571	0.801	-	3.879	0.854	-	6.411

Note: - indicates that the corresponding literature does not give the results for this indicator. Sen represents sensitivity. The measurement unit of ${\mathrm{HD}}_{95}$ is millimeters (mm).

**Table 2:** Comparison of the experimental results on the BraTS 2018 dataset.

Methods	ET		WT		TC		Average	
	DC	HD_95_	DC	HD_95_	DC	HD_95_	DC	HD_95_
Zhou et al. [40]	0.811	2.88	0.908	4.88	0.858	6.93	0.859	4.90
Wang et al. [41]	0.797	3.31	0.902	6.18	0.858	6.37	0.853	5.23
Isensee et al. [42]	0.807	2.74	**0.909**	5.83	0.852	7.20	0.856	5.26
Myroneko et al. [43]	0.815	3.81	0.904	**4.48**	0.860	8.28	0.859	5.52
Our	**0.818**	**2.72**	0.905	5.64	0.857	**6.72**	**0.860**	**4.87**

Note: The measurement unit of ${\mathrm{HD}}_{95}$ is millimeters (mm). The model methods that achieved the best performance indicators are shown in bold.

**Table 3:** Comparison of the experimental results on the BraTS 2015 dataset.

Methods	ET			WT			TC			Average		
	DC	PPV	Sen	DC	PPV	Sen	DC	PPV	Sen	DC	PPV	Sen
Chen et al. [44]	0.61	0.66	0.63	0.85	0.86	0.86	0.72	0.83	0.68	0.72	**0.78**	0.72
Kamnitsas et al. [45]	0.63	0.63	0.67	0.85	0.85	0.88	0.67	**0.86**	0.60	0.71	**0.78**	0.71
Sun et al. [46]	0.62	0.60	**0.69**	0.84	0.82	0.89	0.72	0.77	0.73	0.72	0.73	0.77
Pereira et al. [47]	0.62	0.60	0.68	0.84	0.85	0.86	0.72	0.82	0.76	0.72	0.75	0.76
Isensee et al. [42]	0.64	**0.72**	0.60	0.85	**0.91**	0.83	0.74	0.73	**0.80**	0.74	**0.78**	0.74
Our	**0.65**	0.64	0.68	**0.86**	0.87	**0.90**	**0.75**	0.82	0.78	**0.75**	0.77	**0.78**

Note: Sen represents sensitivity. The model methods that achieved the best performance indicators are shown in bold.

**Table 4:** Experimental results showing the effectiveness of the MMN, SMN, and DRM in different combinations.

Methods	ET				WT				TC			
	DC	PPV	Sen	HD_95_	DC	PPV	Sen	HD_95_	DC	PPV	Sen	HD_95_
MMN	0.814	0.795	0.834	4.461	0.891	0.948	0.868	7.904	0.855	0.920	0.841	5.370
MMN & Flair	0.817	0.798	0.827	3.599	0.896	0.940	0.883	8.129	0.867	0.892	0.851	5.362
MMN & T1c	0.823	**0.811**	0.835	3.897	0.904	0.952	0.873	7.524	**0.885**	0.952	0.848	**4.629**
MMN & SMN	0.821	0.795	0.861	3.615	0.909	**0.953**	0.879	8.130	0.879	**0.957**	0.855	5.321
NoDRM	0.815	0.876	0.876	3.474	0.913	0.945	0.884	7.727	0.860	0.925	0.842	4.764
SR	0.832	0.788	0.880	3.404	0.918	0.932	0.901	7.666	0.876	0.929	0.826	5.836
CR	0.868	0.775	0.882	3.478	0.916	0.947	0.891	7.535	0.868	0.924	0.850	4.807
MMN & SMN & DRM	**0.898**	0.807	**0.893**	**3.298**	**0.923**	0.945	**0.920**	**7.508**	0.879	0.948	**0.866**	5.109

Note: Sen represents sensitivity. The measurement unit of ${\mathrm{HD}}_{95}$ is millimeters (mm). The model methods that achieved the best performance indicators are shown in bold.

## Data Availability

The original data presented in the study are openly available in [16,39].
